# *Rab-3* and *unc-18* Interactions in Alcohol Sensitivity Are Distinct from Synaptic Transmission

**DOI:** 10.1371/journal.pone.0081117

**Published:** 2013-11-14

**Authors:** James R. Johnson, Sudhanva Kashyap, Kim Rankin, Jeff W. Barclay

**Affiliations:** Department of Cellular and Molecular Physiology, The Physiological Laboratory, Institute of Translational Medicine, University of Liverpool, Liverpool, United Kingdom; The University of Queensland, Australia

## Abstract

The molecular mechanisms underlying sensitivity to alcohol are incompletely understood. Recent research has highlighted the involvement of two presynaptic proteins, Munc18 and Rab3. We have previously characterised biochemically a number of specific Munc18 point mutations including an E466K mutation that augments a direct Rab3 interaction. Here the phenotypes of this and other Munc18 mutations were assessed in alcohol sensitivity and exocytosis using *Caenorhabditis elegans*. We found that expressing the orthologous E466K mutation (*unc-18* E465K) enhanced alcohol sensitivity. This enhancement in sensitivity was surprisingly independent of *rab-3*. In contrast *unc-18* R39C, which decreases syntaxin binding, enhanced sensitivity to alcohol in a manner requiring *rab-3*. Finally, overexpression of R39C could suppress partially the reduction in neurotransmitter release in *rab-3* mutant worms, whereas wild-type or E465K mutants showed no rescue. These data indicate that the epistatic interactions between *unc-18* and *rab-3* in modulating sensitivity to alcohol are distinct from interactions affecting neurotransmitter release.

## Introduction

Drug addiction is one of the leading causes of preventable death, generating a considerable financial burden to society. Indeed alcohol use and abuse can lead to increased incidence of liver disease, cardiovascular disease, cancer and other debilitating illnesses [1]. Although the environment can influence addiction, current estimates of genetic heritability range between 40-80% [2]. One significant contributing component to the genetic determination of addiction is the individual’s initial level of response as highlighted by a consistent association of alcohol addiction with polymorphisms in genes involved in alcohol metabolism [3,4]. Despite a ubiquitous prevalence in modern society, the precise physiological mechanisms of intoxication and addiction remain poorly understood. A complete understanding of the contributing factors that underlie alcohol sensitivity is therefore of potential therapeutic importance.

 Current models of alcohol action within the nervous system predict low-affinity interactions of alcohol with specific target proteins or protein complexes [5]. Genetic studies of alcohol sensitivity have contra-indicated many potential targets both pre- and post-synaptic in origin [6,7]. The model organism *Caenorhabditis elegans* is an excellent platform for the genetic dissection of alcohol sensitivity as it has a similar dose-dependent response to exogenous alcohol to mammals [8]. Recent research from *C. elegans* has determined a role in alcohol sensitivity for proteins central to the exocytotic machinery, yet distinct from synaptic transmission efficacy. *Loss-of-function (lof*) mutations in the GTPase *rab-3* reduces sensitivity to alcohol in *C. elegans* [9]. Similarly, a single point mutation in the protein Munc18 that inhibits SNARE complex binding specifically also reduces sensitivity to alcohol in *C. elegans* [10]. Both mutants also affect voluntary alcohol consumption in mice [9,11] emphasising the conservation of genetic determination of alcohol sensitivity from nematodes to mammals. 

 Munc18 is an essential protein in presynaptic vesicle exocytosis whose precise function remains somewhat enigmatic [12,13]. Biochemically, Munc18 binds the t-SNARE (soluble N-ethylmaleimide-sensitive factor attachment protein receptor) syntaxin in two different modes of interaction as well as the assembled SNARE complex [14-16]. In worms, null *unc-18* (*e81*) alleles display strong behavioural phenotypes including paralysed locomotion and resistance to inhibitors of acetylcholinesterase [17]. Rab3 is a GTPase that also functions in exocytosis by recruiting and tethering synaptic vesicles to the plasma membrane [18], although roles for Rab3 in late stages of docking [19] and vesicle fusion [20] have also been demonstrated. In worms, *lof rab-3* mutants exhibit loopy, mildly slower locomotion and are also resistant to inhibitors of acetylcholinesterase [19].

 We have previously investigated a number of point mutations of mammalian Munc18 that alter protein interactions [21], including an E466K *gain-of-function (gof*) mutation affecting direct binding to Rab3 [22]. In this study we have investigated the functional effects of some of these point mutations in *unc-18*, the nematode orthologue of Munc18-1, in both a wild-type and *lof rab-3* genetic background. A mutation that interferes with closed-conformation syntaxin binding (*unc-18* R39C) was hypersensitive to alcohol as was the orthologous mutation that enhances the Munc18-Rab3 interaction (*unc-18* E465K). In addition overexpression of the R39C mutation partially compensated for *lof rab-3* in neurotransmitter release; yet, was recessive to *lof rab-3* in alcohol sensitivity. Conversely, the E465K mutation was dominant to *lof rab-3* in alcohol sensitivity, but recessive in neurotransmitter release. We conclude that the specific interactions between *unc-18* and *rab-3* that govern exocytosis are functionally distinct from sensitivity to alcohol.

## Results

### Alcohol sensitivity phenotypes of single point mutations in *unc-18*

**Figure 1:**
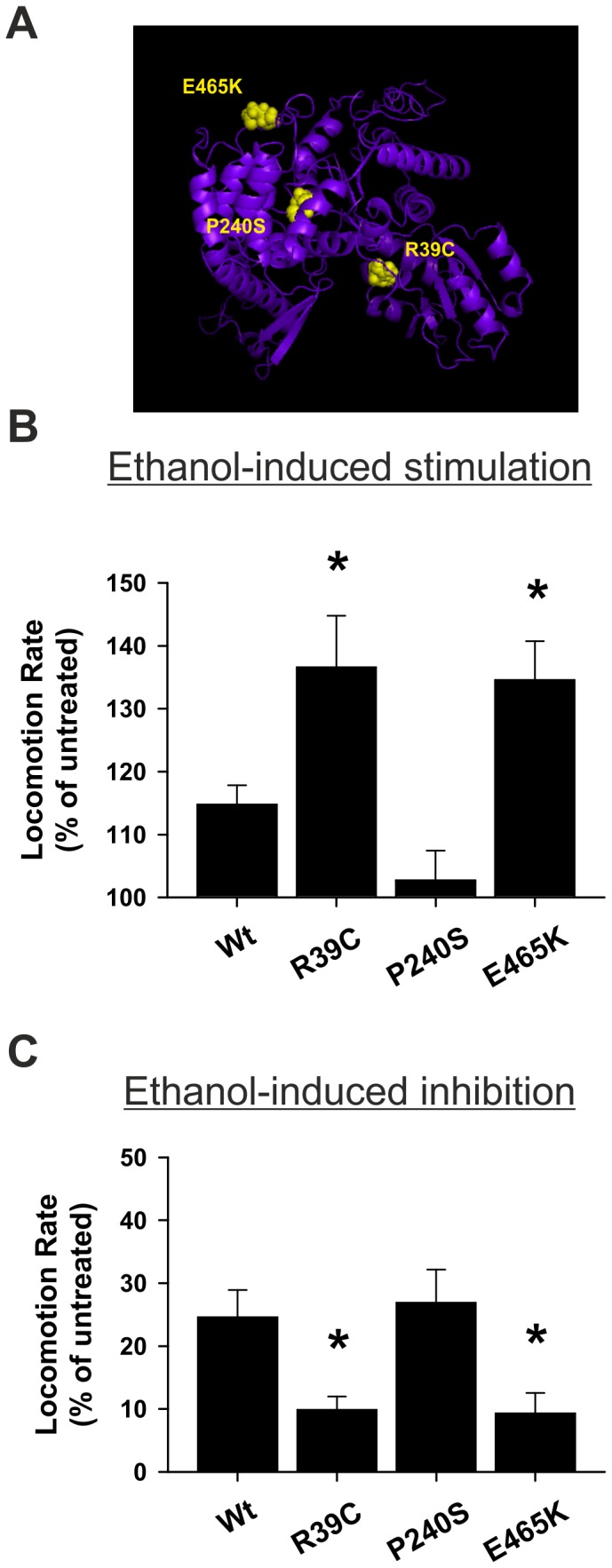
Expression of *unc-18* R39C or E465K point mutations enhances acute alcohol sensitivity. (A) Location of the three investigated point mutations of *unc-18* (R39C, E465K and P240S) in a predicted model of UNC-18 structure. Null *unc-18* (*e81*) worms were rescued transgenically with either wild-type (Wt) *unc-18* or *unc-18* with the indicated point mutations. In comparison to Wt *unc-18*, both the R39C and E465K point mutations increased acute sensitivity to ethanol at (B) stimulatory levels (21 mM) and (C) depressive levels (400 mM) of external ethanol. For (A) significance was assessed by one-way analysis of variance with post-hoc comparisons; *P<0.05; N = 28 (Wt), 20 (R39C), 22 (P240S) and 20 (E465K). For (B) significance was assessed by Kruskal-Wallis one-way analysis of variance with post-hoc comparisons; *P<0.05; N = 29 (Wt), 40 (R39C), 20 (P240S), 18 (E465K).

 We recently demonstrated that a single point mutation (D216N) in Munc18 acts biochemically by reducing binding to the assembled SNARE complex and that the orthologous mutation in *C. elegans unc-18* (D214N) reduces sensitivity to both low and high concentrations of exogenous ethanol [10]. Previously, we have biochemically characterised other point mutations in Munc18 that affect binding to other proteins including R39C (inhibits binding to closed-conformation syntaxin) [23,24], P242S (inhibits binding to Mint proteins) [21] and E466K (enhances binding to Rab3) [22]. To assess whether these other Munc18 interactions could also affect alcohol sensitivity we generated transgenic worms expressing the orthologous mutations of *unc-18* in a null (*unc-18 e81*) background (Figure 1A) and assessed their sensitivity to alcohol in comparison with transgenic worms expressing wild-type *unc-18*. Despite a strong reduction in alcohol sensitivity, worms that express the *unc-18* D214N mutation have relatively normal, but statistically elevated locomotion rates [10]. Similarly, the *unc-18* R39C, P240S and E465K expressing mutants exhibited qualitatively normal locomotion in comparison to *unc-18* wild-type (Table 1) although the R39C mutants had a significant reduction in thrashing of 23% in comparison to wild-type (Kruskal-Wallis one-way analysis of variance on ranks with post-hoc comparison; P<0.05; N = 77 (Wt), 55 (R39C), 48 (P240S) and 55 (E465K)). 

**Table 1:** Basal locomotion rates of *C. elegans* strains used in this study.

*C. elegans* strain	**Locomotion Rate(thrashes/min)**
Bristol N2	88.7±2.5
*unc-18* (*e81*) + *unc-18* Wt	67.5±2.6
*unc-18* (*e81*) + *unc-18* R39C	51.9±2.1
*unc-18* (*e81*) + *unc-18* P240S	69.6±2.6
*unc-18* (*e81*) + *unc-18* E465K	61.5±3.9
*unc-18* (*e81*) + *unc-18* R39C/E465K	79.3±1.4
*rab-3 (y250*)	78.9±2.0
*rab-3* (*y250*) + *unc-18* Wt	72.6±1.6
*rab-3* (*y250*) + *unc-18* R39C	60.9±2.1
*rab-3* (*y250*) + *unc-18* E465K	65.2±3.0
*rab-3* (*y250*) + *unc-18* R39C/E465K	70.1±2.4

 Exposing worms to high external ethanol concentrations (400 mM) causes a depression in locomotion [8,25]. In addition, exposure of worms to low external concentrations (21 mM) stimulates locomotion [10]. Due to the low permeability of chemicals across the *C. elegans* cuticle, the internal ethanol concentrations are estimated to be substantially reduced and approximate that seen in intoxicated humans [8], although this interpretation is not universally shared [25]. We screened whether any of the *unc-18* point mutations had effects on sensitivity to exogenous ethanol at either the stimulatory or depressive concentrations. In contrast to the previously characterised D214N mutation, the R39C and E465K mutations enhanced sensitivity to alcohol at both the stimulatory and the depressive concentrations (Figure 1B, C). There were no effects of the P240S mutation at either concentration of ethanol. This lack of effect was perhaps unsurprising as the P240S mutation reduces binding to the Mint proteins [21] and the *C. elegans* orthologue of Mint, *lin-10*, lacks the Munc18 binding domain. Therefore, both the R39C and E465K mutations of *unc-18* increased sensitivity to alcohol.

### Alcohol sensitivity phenotype of a double mutation in *unc-18*

**Figure 2:**
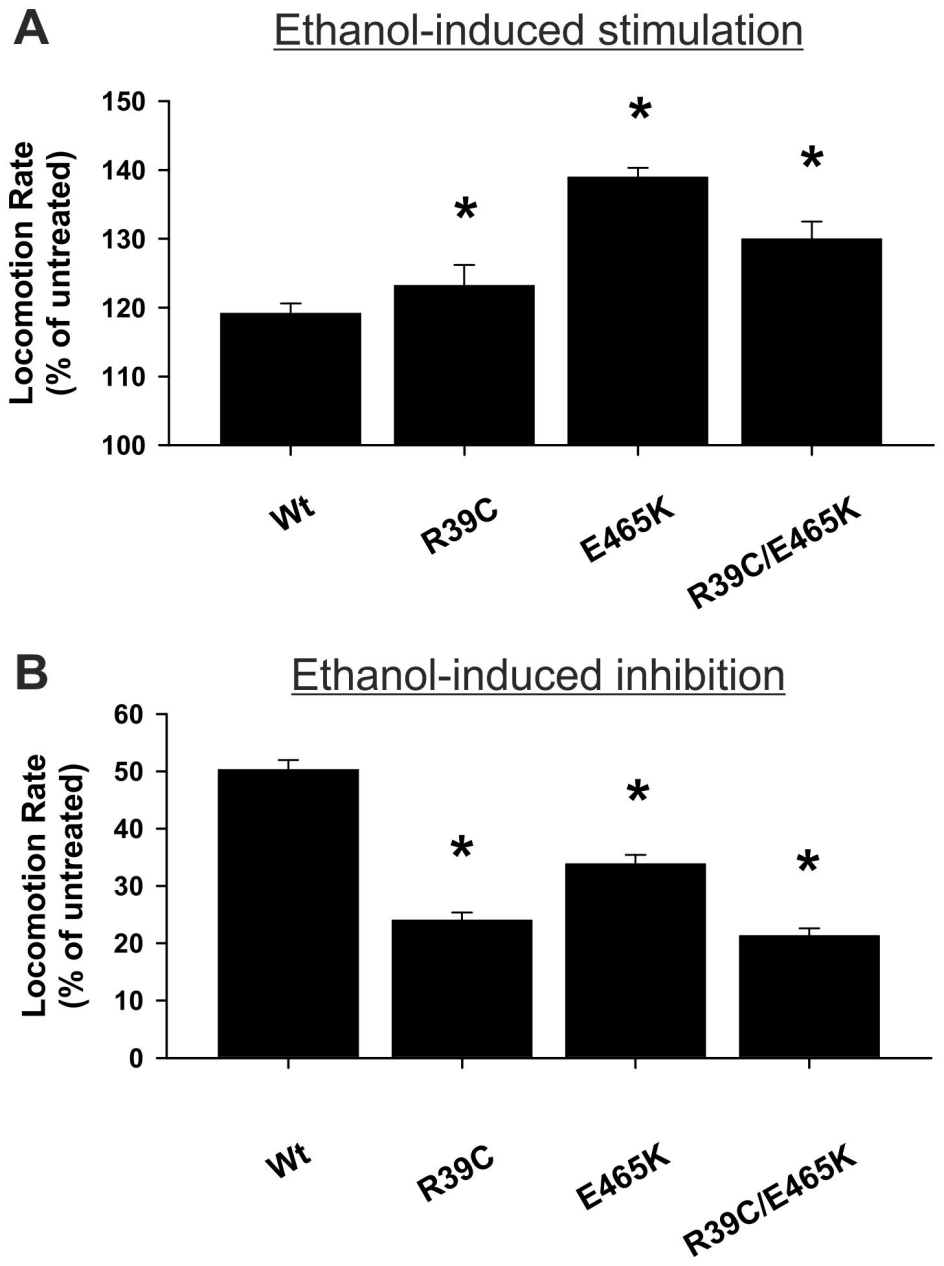
The phenotypic effects of *unc-18* R39C and E465K in alcohol sensitivity are not additive. Null *unc-18* (*e81*) worms were rescued transgenically with wild-type (Wt) *unc-18* or *unc-18* with the indicated point mutations. In comparison to Wt *unc-18*, all three mutants had an increase in acute sensitivity to ethanol at (A) stimulatory levels (21 mM) and (B) depressive levels (400 mM) of external ethanol. For (A) significance was assessed by Kruskal-Wallis one-way analysis of variance with post-hoc comparisons; *P<0.05; N = 15 (Wt), 15 (R39C), 15 (E465K) and 15 (R39C/E465K). For (B) significance was assessed by one-way analysis of variance with post-hoc comparisons; *P<0.05; N = 15 (Wt), 15 (R39C), 15 (E465K), 15 (R39C/E465K).

 Munc18 functions at the synapse at multiple steps in the exocytotic pathway through interactions with many proteins [12,13]. We were interested to determine whether the enhanced sensitivity to alcohol of the R39C or E465K mutations had additive phenotypic effects when combined. To assess this question, we generated transgenic worms expressing the double mutation (*unc-18* R39C/E465K). Although the single mutants each had small inhibitory effects on basal thrashing rate, locomotion of the double mutant was in fact enhanced to a greater level than wild-type (Table 1; Kruskal-Wallis one-way analysis of variance on ranks with post-hoc comparisons; P<0.05; N = 55 (R39C), 55 (E465K) and 15 (R39C/E465K)). In comparison to worms expressing wild-type *unc-18* or either single mutants, however, the double mutation (R39C/E465K) produced no additive effect on alcohol sensitivity (Figure 2A, B). At either low or high external ethanol the sensitivity of the double mutant was not significantly greater than the single mutants. Therefore the effects of either point mutation were not additive with respect to alcohol sensitivity.

### Exocytotic phenotypes of point mutations in *unc-18*

 Movement of nematodes is determined by defined neural circuits, integrating sensory information to generate locomotion, as well as the strength of neuromuscular transmission. Munc18 has primarily been described as a protein essential for exocytosis [12,13]. Mice null for Munc18 have defects in both vesicle docking [26] and secretion [27]. In addition specific mutations of Munc18 can affect the kinetics of membrane fusion [15,28,29]. In *C. elegans*, *unc-18* null mutants are paralysed and have defects in docking and neuromuscular transmission [17,30]. With respect to exocytosis, the R39C mutation causes an increase in EJP amplitude in *Drosophila* [31] and alters the kinetics of vesicle fusion in chromaffin cells [24], whereas it appears to have very little effect when expressed in *C. elegans* [23,32]. The E466K mutation enhances dense-core granule recruitment in chromaffin cells [22] and has a very mild hypersensitivity to aldicarb in *C. elegans* [33]. 

**Figure 3:**
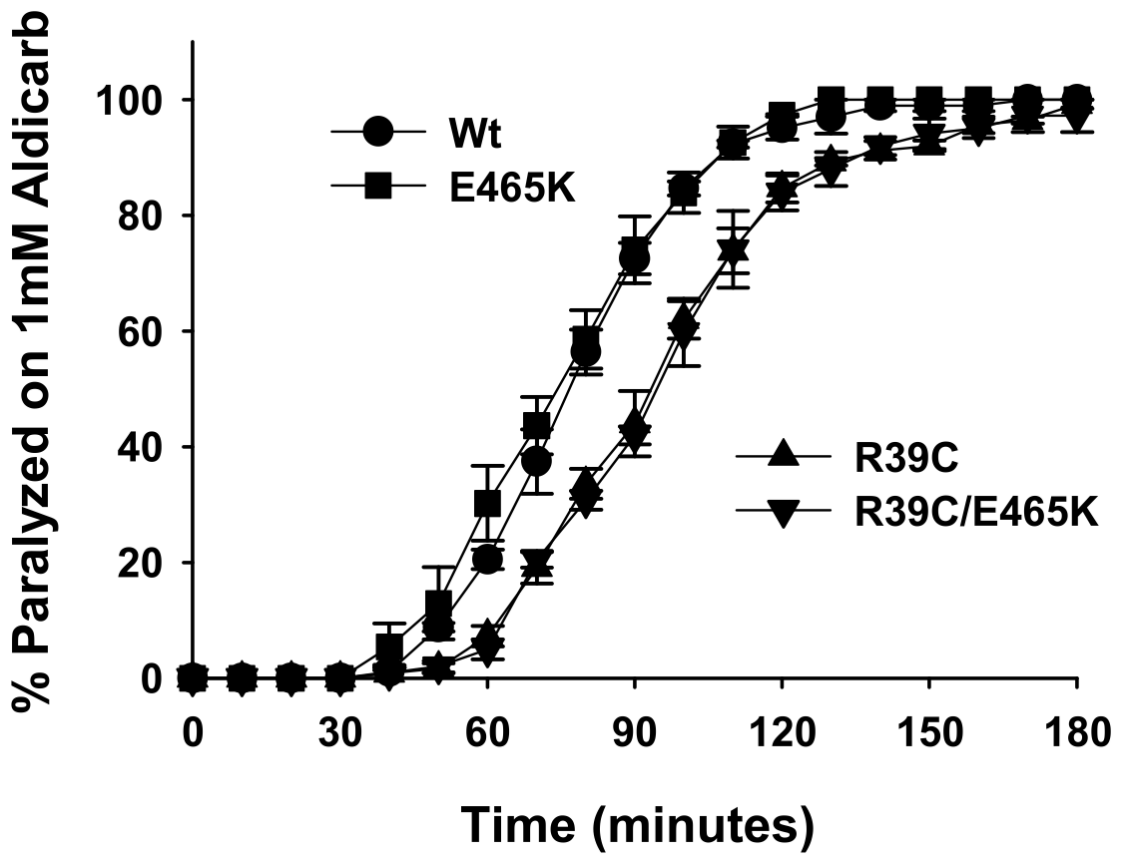
R39C *unc-18* mutants are resistant to aldicarb. Null *unc-18* (*e81*) worms were rescued transgenically with wild-type (Wt) *unc-18* or *unc-18* with the indicated mutations. The aldicarb sensitivity of the R39C and R39C/E465K mutants were significantly decreased in comparison to Wt *unc-18*. Significance was assessed by two-way analysis of variance with post-hoc comparisons. R39C (P<0.001) and R39C/E465K (P<0.001) were different from Wt, but not from each other (P=0.71). N = 3 experiments per strain (20-25 worms each experiment).

 We next determined whether any of the mutations in *unc-18* that affected alcohol sensitivity also affected the strength of synaptic transmission using the well established aldicarb sensitivity assay [34]. In this assay, quantitative changes in the rate at which a population of worms paralyse are indirect measurements of changes in synaptic strength. In comparison to worms expressing wild-type *unc-18*, the E465K mutants were mildly, but insignificantly, hypersensitive to aldicarb (Figure 3). In contrast, R39C worms had a small, but consistent resistance to aldicarb indicative of a reduction in signalling strength at the neuromuscular junction. We also tested the R39C/E465K double mutant in the aldicarb assay and found that the R39C mutation was dominant over E465K for the aldicarb sensitivity phenotype (Figure 3). As the two *unc-18* mutations produced equivalent effects in ethanol but contrasting effects in aldicarb, we conclude that the function of the individual mutations in sensitivity to alcohol are uncorrelated with effects on synaptic transmission strength.

### Alcohol sensitivity phenotypes of point mutations in *lof rab-3*

 Rab3 is a GTPase involved in the trafficking of synaptic vesicles and various aspects of exocytosis [18]. *Lof rab-3* worms are resistant to the effects of depressive concentrations of exogenous alcohol [9]. The E466K mutation of Munc18 increases the interaction between Munc18 and Rab3 [22] without affecting binding to syntaxin or Mint proteins [21]. We therefore investigated whether the effects of any of our *unc-18* mutations were epistatic to *rab-3* by expressing in a *lof rab-3* genetic background and assaying for alcohol sensitivity. We have previously investigated the effects of specific *unc-18* point mutations in both a wild-type (N2) or null (*unc-18*) genetic background and found similar phenotypic effects either in the presence or absence of endogenous *unc-18* [35]. Similar to that seen in the null *unc-18* (*e81*) allele, expression of R39C in *lof rab-3* (*y250*) caused a significant decrease in basal locomotor rate in comparison to expression of wild-type *unc-18* (Table 1; one-way analysis of variance with post-hoc comparison; P<0.05; N = 25 (*rab-3 y250*), 25 (Wt), 30 (R39C) and 30 (E465K)). The enhancement in locomotor rate by the R39C/E465K double mutant in comparison to the single R39C mutation was also apparent in the *lof rab-3* background (one-way analysis of variance with post-hoc comparison; P<0.05; N = 30 (R39C), 30 (E465K) and 30 (R39C/E465K)).

**Figure 4:**
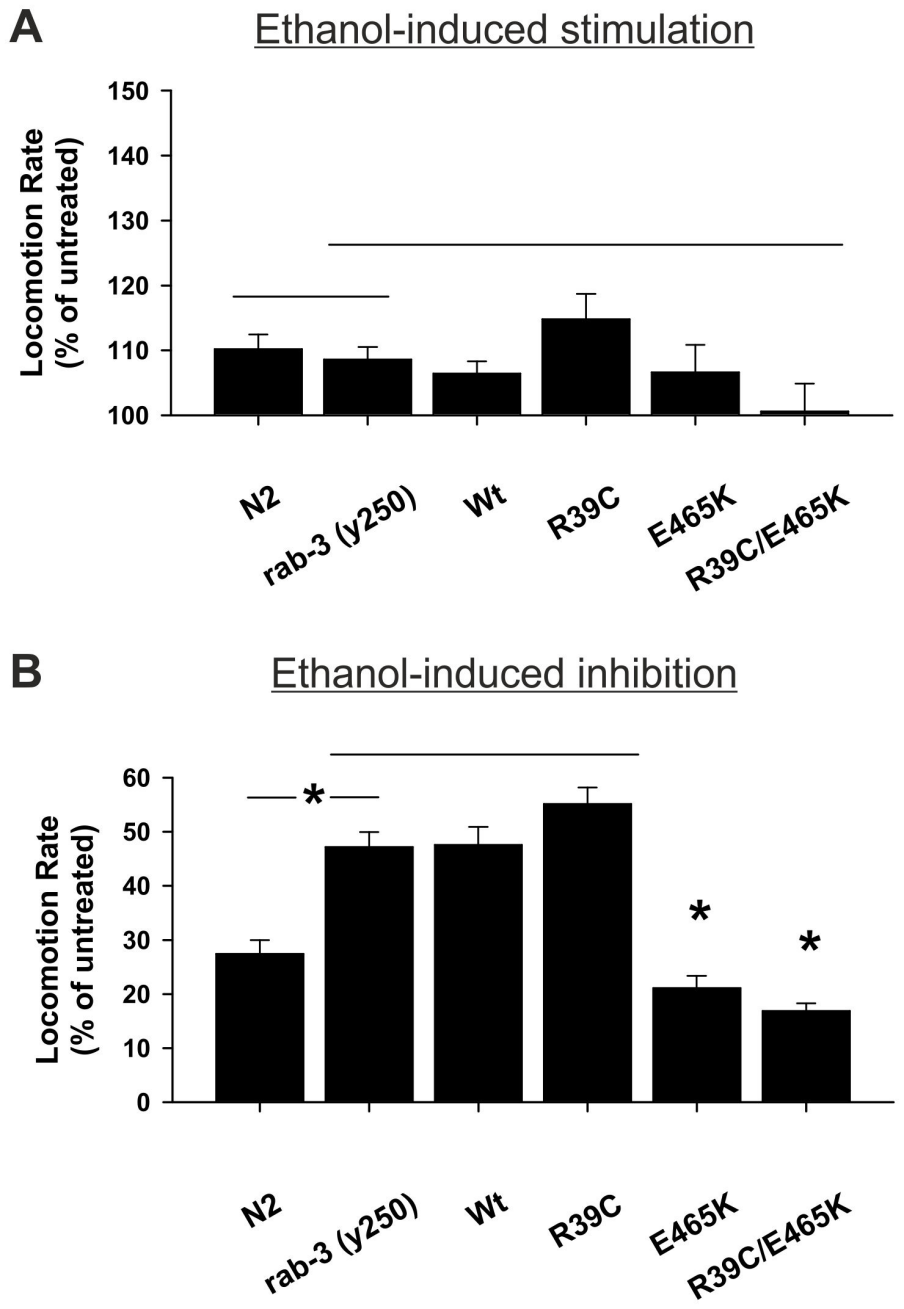
The E465K *unc-18* point mutation suppresses the *rab-3*-dependent resistance to alcohol. *Loss-of-function *(*lof*) *rab-3* (*y250*) worms were created to express transgenically wild-type (Wt) *unc-18* or *unc-18* with the indicated mutations. (A) Expression of either Wt or mutant *unc-18* in a *lof* *rab-3* genetic background did not alter acute sensitivity to external ethanol at stimulatory levels (21 mM). Significance was assessed by one-way analysis of variance; P=0.46; N = 29 (N2), 37 (*rab-3*), 31 (Wt), 31 (R39C), 31 (E465K) and 31 (R39C/E465K). (B) In comparison to Bristol N2 controls, *lof* *rab-3* worms had decreased acute sensitivity to external ethanol at inhibitory levels (400 mM). Expression of either *unc-18* E465K or R39C/E465K, but not R39C, was dominant to *rab-3*. Significance was assessed by Kruskal-Wallis one-way analysis of variance on ranks with post-hoc comparisons; *P<0.05; N = 33 (N2), 40 (*rab-3*), 30 (Wt), 31 (R39C), 31 (E465K) and 31 (R39C/E465K).

 In response to low levels of alcohol, *lof rab-3* worms exhibited a normal stimulation of locomotion (Figure 4A). The enhancement in alcohol-dependent stimulation by either single (R39C or E465K) or double (R39C/E465K) mutations of *unc-18*, however, was negated when these mutations were expressed in the *lof rab-3* mutant background. As previously described [9], at depressive concentrations of ethanol *lof rab-3* (*y250*) worms were less sensitive than Bristol N2 wild-types (Figure 4B). Expressing either wild-type (Wt) or R39C *unc-18* in *lof rab-3* had no effect on this *rab-3* phenotype. Surprisingly, expression of *unc-18* E465K was dominant to the effects of *lof rab-3*. Expression of the double mutant showed that the addition of the R39C mutation did not alter the dominant effect of E465K (Figure 4B). These experiments demonstrate that at low concentrations of ethanol, the *lof rab-3* phenotype is dominant to both of the R39C and E465K *unc-18* mutations whereas at high concentrations, E465K is dominant to *rab-3* whereas *rab-3* is dominant to R39C.

### Exocytotic phenotypes of point mutations in *lof rab-3*

**Figure 5:**
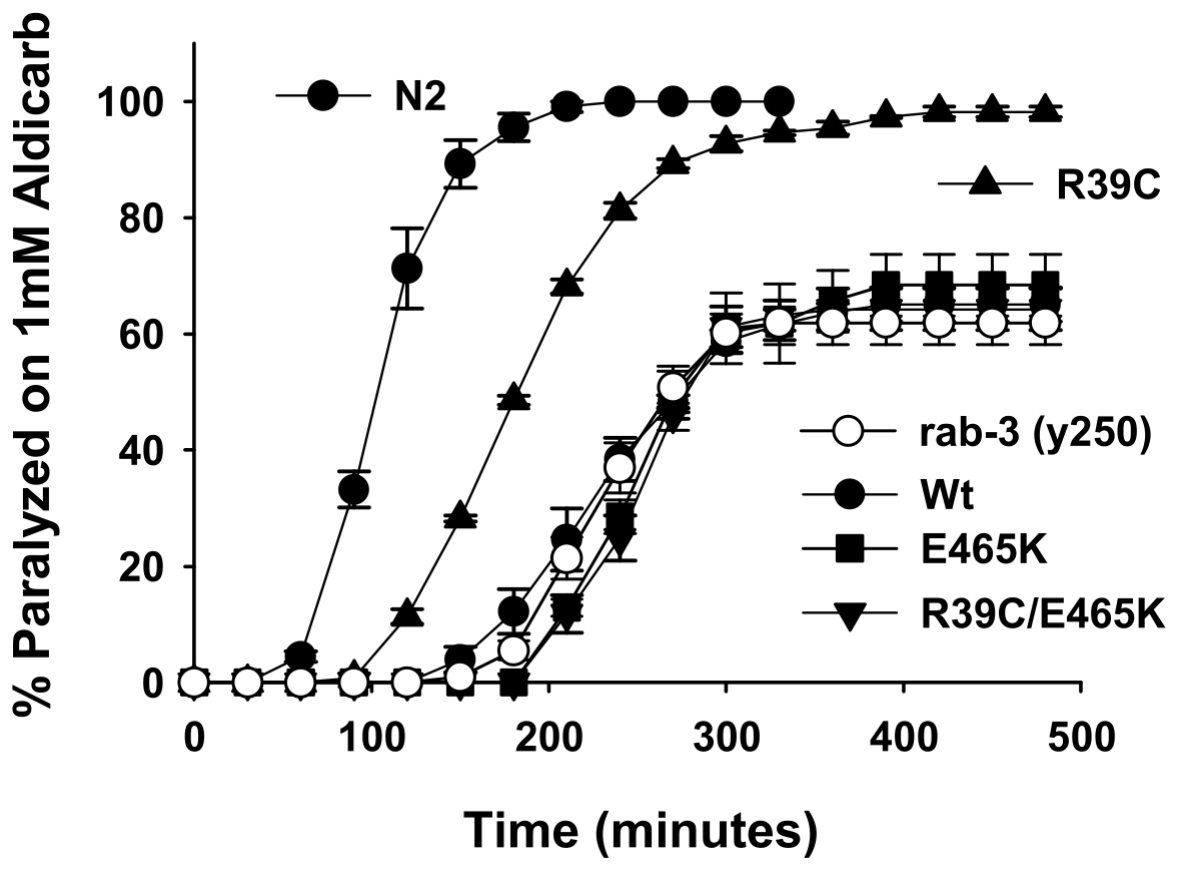
The R39C *unc-18* point mutation suppresses the *rab-3*-dependent resistance to aldicarb. *Loss-of-function *(*lof*) *rab-3* (*y250*) worms were created to express transgenically wild-type (Wt) *unc-18* or *unc-18* with the indicated mutations. In comparison to Bristol N2 worms, *lof* *rab-3* worms exhibited resistance to aldicarb. This resistance to aldicarb was blocked partially by expression of R39C *unc-18*. The R39C/E465K double mutation did not affect *rab-3* aldicarb sensitivity. Significance was assessed by two-way analysis of variance with post-hoc comparisons. Only worms expressing R39C (P<0.001) had altered aldicarb sensitivity in comparison to *rab-3* worms. N = 3 experiments per strain (20-25 worms each experiment).

 The E466K mutation enhances the interaction between Munc18 and Rab3 [22], without affecting syntaxin binding [21]. Despite this biochemical characterisation, the effect of the mutation on sensitivity to high concentrations of alcohol was surprisingly independent of functional *rab-3*. We tested whether any of the *unc-18* mutations required *rab-3* to affect exocytosis. We verified that *lof rab-3* (*y250*) worms were resistant to aldicarb in comparison to Bristol N2 wild-types (Figure 5) as has been previously reported [19]. Expression of wild-type *unc-18* in the *lof rab-3* background had no effect on *rab-3*-dependent resistance to aldicarb. Despite dominant effects to *lof rab-3* in alcohol sensitivity the E465K mutation had no effect on the aldicarb phenotype. The *unc-18* R39C mutation, which on its own caused a mild resistance to aldicarb, was able to block partially the effects of *lof rab-3* (Figure 5). Thus, despite *lof rab-3* being dominant to R39C in sensitivity to alcohol the reverse was true for sensitivity to aldicarb. The R39C/E465K double mutant was not different from *lof rab-3* indicating that the effects of R39C alone were suppressed by the additional E465K mutation.

## Discussion

 This paper demonstrates that the genetic interactions between two exocytotic proteins, *unc-18* and *rab-3*, are different depending on the phenotypic context. For the alcohol phenotype, either the R39C or E465K *unc-18* mutations increased sensitivity. The R39C mutation is characterised to decrease binding to closed conformation syntaxin for mammalian Munc18 *in vitro* [24] and *in vivo* [36] as well as *C. elegans* UNC-18 *in vitro* [23]. This then potentially implicates this interaction with syntaxin as an important regulator of alcohol sensitivity. Although this hypothesis has not been directly tested for ethanol specifically, syntaxin hypomorphs in *C. elegans* do have reduced sensitivity to volatile anaesthetics [37] emphasizing a potential convergence of cellular effectors of various anaesthetics at the presynaptic terminal. On the other hand, the E465K mutation acts to increase Rab3 binding, at least for Munc18 [22]. Applying the same logic of R39C and syntaxin, this would imply that increased ethanol sensitivity of the E465K mutation would be a consequence of increased Rab3 binding. Rab3 itself does not associate with Munc18 when it is syntaxin bound [22]. Consequently the ethanol phenotype of E465K alternatively could be a secondary consequence of the reduction in syntaxin binding in favour of Rab3. This interpretation could also explain the lack of additivity of the double mutant. 

 The results of these mutations in the *lof rab-3* genetic background, however, argue against the simple interpretation that the effects are solely the result of the same syntaxin interaction. For the stimulatory ethanol phenotype, the effects of R39C or E465K mutations were blocked. For the depressive ethanol sensitivity phenotype the E465K mutation is dominant to *lof rab-3* whereas R39C is not. This then indicates that whatever the E465K mutation is doing at high ethanol concentrations, it acts both downstream and independent of functional *rab-3*, which itself is downstream of R39C. Interestingly, the E465K mutation is modelled on a Sly1p (yeast Sec1/Munc18 protein) that bypasses the requirement for a functional Rab protein during ER to Golgi vesicle trafficking [38]. This mutation then also bypasses the requirement of a functional Rab protein in alcohol sensitivity as expression of E465K in the *lof rab-3* genetic background eliminates the *rab-3* phenotype. What then are these *unc-18* mutations or *lof rab-3* doing to alter ethanol sensitivity? Previous work has excluded the interpretation that ethanol sensitivity is a simple reflection of alterations in signalling strength [8-10]; yet, both *unc-18* and *rab-3* are characterised primarily as exocytotic proteins involved potentially in docking, priming and fusion itself [13,18]. It remains possible that the action of ethanol presynaptically is at the level of synaptic vesicle trafficking or exocytosis that is separate from signalling strength per se. Alternatively, the action of ethanol could be postsynaptic and *lof rab-3* or the *unc-18* mutations are altering the trafficking of postsynaptic receptors whose function is modulated by ethanol. Indeed, ethanol can affect many neurotransmitter receptors including GABA (γ-aminobutyric acid), glutamate and serotonin [7]. The precise synaptic location of action of ethanol and the roles of exocytotic proteins therefore remains to be determined in greater detail. Despite this, it is clear that the *unc-18* E465K mutation acts independently and can circumvent the requirement of functional *rab-3* in ethanol sensitivity.

 The epistatic interactions between *unc-18* and *rab-3* that determine ethanol sensitivity stand in direct contrast to those for signalling strength. At the worm neuromuscular junction, the R39C mutation induced resistance to aldicarb implying a reduction in signalling strength. The R39C mutation has been previously shown to increase evoked postsynaptic currents in *Drososphila* [31] which may be a result of an increase in initial fusion rate [28]. The total amount of neurotransmitter released per exocytotic event, however, is concurrently decreased by the R39C mutation in bovine adrenal chromaffin cells [24] which would explain the observed reduction in signalling strength as assayed by aldicarb sensitivity in *C. elegans*. Contrary to ethanol sensitivity, R39C *unc-18* is partially dominant to *lof rab-3*. Indeed as the R39C mutation is itself resistant to the effects of aldicarb in comparison to wild-type *unc-18*, it is possible that R39C is completely dominant to *lof rab-3* for aldicarb sensitivity. It is most likely that this mutation overcomes the loss of functional *rab-3* in exocytosis via changes to vesicle recruitment. Null *unc-18* worms have a reduction in docked vesicles [30] that is dependent on syntaxin binding [39] and *lof rab-3* alleles also reduce both the total number of synaptic vesicles and their trafficking [19]. Indeed the role of Munc18 in docking is downstream of Rab3 in adrenal chromaffin cells [40]. The data here support the notion that inhibiting the closed-conformation syntaxin interaction, and hence supporting binding of Munc18/UNC-18 to open syntaxin, helps to bypass partially the requirement of Rab3 in determining strength of neurotransmitter release.

 The Munc18 E466K mutation acts to increase Rab3 binding and the number of fusion events from bovine adrenal chromaffin cells [21]. Therefore, the lack of effect of the orthologous mutation (*unc-18* E465K) in the *lof rab-3* genetic background could be relatively easy to rationalise. Indeed the *rab-3* (*y250*) allele produces no detectable RAB-3 protein [19]. The phenotypic effect of the R39C mutation, however, is blocked in the R39C/E465K double mutant expressed in the *lof rab-3* genetic background suggestive of an additional functional role of the E465K mutation. At present, no other biochemical effects of the E465K mutation are known [21,22]. Nonetheless, in contrast to ethanol sensitivity, the aldicarb data indicate that that the R39C mutation acts downstream and independently of *rab-3*, which itself is potentially downstream of E465K.

 The phenotypic effects presented here are likely to be consistent with phenotypic effects in mammals. Indeed, the pleiotropic action of alcohol in mammals is conserved for many phenotypes in nematodes [41]. Mutations that affect ethanol sensitivity in nematodes have been consistently demonstrated to alter more complex alcohol phenotypes in mice, including Munc18 and Rab3 [8-11,42,43]. In fact, various GTPases have been linked with addiction in general [44-47]. Whether Munc18/UNC-18 itself is acting as an effector of Rab3 is a potential hypothesis requiring more investigation. For the exocytosis phenotypes, key insights have been derived from *C. elegans*, as the vast majority of exocytotic proteins have orthologues in nematodes [48]. The interactions between Munc18/UNC-18 and Rab3 have thus far been only investigated with respect to exocytosis [22,40,49] and this study furthers this knowledge by showing that the *unc-18* R39C mutation can overcome the secretory defects associated with *lof rab-3*. In addition, genetic interactions between *unc-18* and *rab-3* in alcohol sensitivity determined that, for this phenotype, the *unc-18* E465K mutation eliminated a requirement of *rab-3*. Most surprisingly, we demonstrate that the epistatic interactions between mutants of *unc-18* and *rab-3* are distinct depending on the phenotypic context such that the R39C mutation acts downstream of Rab3 in exocytosis whereas it acts upstream of Rab3 in ethanol sensitivity. Finally, our data emphasises that simple modulation of synaptic strength is unrelated to sensitivity to ethanol and that the functional actions of alcohol are a complex cellular mechanism involving a large spectrum of neuronal proteins. 

## Materials and Methods

### Molecular biology

 All point mutations of the *unc-18* rescuing construct were introduced by site-directed mutagenesis using either the GeneTailor (Invitrogen) or QuikChange (Stratagene) methods as described previously [10,23].

### Nematode culture, strains and microinjection

*C. elegans* strains were grown and maintained on nematode growth medium (NGM) plates at 20°C with *Escherichia coli* OP 50 as a food source as previously described[10,23]. Strains used in this study were: Bristol N2 (wild-type reference), *unc-18* (*e81*) and *rab-3* (*y250*). Transgenic worms were generated by germline injection as previously described [10,23]. Transgenic expression constructs carried *unc-18* cDNA, either wild-type or the indicated point mutations, under the control of its own genomic flanking regions. Successful transgenic expression was verified by co-injection with a *sur-*5::GFP marker (pTG96) (kind gift of Prof. A. Fire, Stanford, CA). The concentration of injected DNA was made up to 100 ng/µl with empty pBlue Script SK+ vector for all injections. For each transgenic construct, 3-5 individual independently-derived lines were generated and analysed. Results presented here were consistent for all generated lines.

### Behavioural assays and analysis

 All behavioural assays were performed in a temperature controlled room at 20°C using young adult hermaphrodite animals from sparsely populated plates. Locomotion rate was quantified by measuring thrashing in 200 ul Dent’s solution (140 mM NaCl, 6 mM KCl, 1 mM CaCl_2_, 1 mM MgCl_2_ and 5 mM HEPES; pH 7.4; with bovine serum albumin at 0.1 mg/ml) over a 1 minute period as described previously [10,23]. A thrash was defined as one complete movement from maximum to minimum amplitude and back again. For ethanol experiments, measurements of locomotion were made after 10 minutes exposure and are expressed as a percentage of mean locomotion rate in 0 mM ethanol measured each day (at least 10 control animals per transgenic line). Animals were assessed in both low ethanol concentrations that stimulate locomotion (21 mM) and high ethanol concentrations that depress locomotion (400 mM) [8,10,25]. All data are expressed as mean ± S.E. Significance was tested by one-way analysis of variance (ANOVA) and post-hoc comparison of means using either the Student-Newman-Keuls test or Dunn’s test (where samples sizes were unequal). Aldicarb sensitivity was determined by measuring time to paralysis following acute exposure. For each experiment, 20-25 worms were moved to NGM plates containing aldicarb (1 mM; Sigma Chemical) and assessed for paralysis every 10 or 30 minutes after drug exposure by mechanical stimulation of the worms with a thin tungsten wire. Significance was tested by two-way ANOVA and post-hoc comparison of means using the Student-Newman-Keuls test. Experiments were performed three times.
